# Student-centered, “embodied inter-referencing” as antiracist and anticolonial pedagogy

**DOI:** 10.1007/s12564-022-09811-3

**Published:** 2022-12-03

**Authors:** Ayaka Yoshimizu

**Affiliations:** Department of Asian Studies, The University of British Columbia, 607-1871 West Mall, Vancouver, BC V6T 1Z2 Canada

**Keywords:** Asia as method, Diaspora as method, Inter-referencing, Embodied narrative, Antiracist pedagogy, Decolonial pedagogy

## Abstract

This article reflects on an experimental pedagogical approach I developed in a Japanese literature course that examines sex, gender, and sexuality in response to institutional and epistemic racism that exist in university in Canada and in the specific context of the COVID-19 pandemic when the number of anti-Asian hate crimes rose at an enormous rate in the city where my university is situated. Building on the intellectual movements of “Asia as method” and “diaspora as method” my project attempts to move beyond the convention of studying Asian culture by referencing western theory, knowledge, and experience. More specifically, I developed an assignment called Peer- Engaged Embodied Reflection Journal where students discuss what they learned from Japanese literature by referencing their own, local experiences and engage in peer interactions in small groups. In this article, I discuss the effectiveness of my pedagogical approach based on the classroom study conducted in the fall of 2020 and the spring of 2021, semi-structured interviews with teaching assistants (TAs), and my own teaching experience. Based on my qualitative analysis of student engagement with the assignment as well as TAs’ and my grading experiences, I conclude that recentring student experience and peer engagement produces meaningful sites for decolonial and antiracist pedagogy while teaching Japanese modern literature in a Canadian institutional context.

## Introduction

As I was reading the student evaluations for my second-year Japanese literature course, Sex, Gender, and Sexuality in Japanese Literature and Film, for fall of 2019, one student’s comment stood out. After pointing out how sex and gender are considerably tied to race from their experience of living in Vancouver, Canada, the student writes, “I hope there is more space for students to discuss what it means to be diasporic Asian females, males, and/or queers in Canada in connection to what we read in the course.” While this comment was expressed as a suggestion for how this course might be taught in the future, it has broader implications. Alongside the decade-long, racist model-minority discourse that claims that some Canadian universities, including the one where I teach, are “too Asian” (Findlay & Köhler, 2010; Huynh, 2020), the comment revealed that the Canadian academy remains fundamentally white at its structural and epistemic levels; that is, whiteness persists as an assumed identity for the ideal student body (Bunjun, 2021a; Coloma, 2017). Against such “normativity of whiteness,” as Kim puts it in her critique of the “too Asian” discourse, is the enduring idea that “here is a kind of invasive Asian presence…foreign and encroaching upon Canadian universities in a kind of space that does not belong to them” (cited in Huynh, 2020, n.p.). In fact, the student’s comment implies that classrooms have not normalized the discussion of Asian identities at the risk of making classes “too Asian.”

The following term saw the outbreak of the COVID-19 pandemic and my university’s transition to online teaching on March 16, 2020. While we continued to teach students remotely in the summer and the 2020/21 academic year, hate crimes against Asians rose at an enormous rate; in October 2020, the Vancouver Police Department reported that such incidents increased more than 878% from the previous year (Kotyk, 2020). My university campus in Vancouver also became a site for hate crimes, which included vandalized posters put up in the science building (Huynh, 2020) and the physical assault of a Korean student in University Village (Hong & Alden, 2021). Although the situation was so overwhelming relative to the impact I could make in my small acts of teaching, something had to be done in my own teaching practice.

Nevertheless, what was striking about the student’s comment in the 2019 course evaluation was not just the lack of campus space to discuss their Asian identities; it also suggested that the student associated their lived experience as an Asian diasporic subject in Canada to stories originally produced in Japan, pointing to a potential intersection between Asian area studies and Asian diaspora studies, which have long been considered as separate fields with an uneasy relation: the former being initially developed as a colonial and Cold War strategy in the West to produce knowledge about their allies and enemies in Asia using the nation-state framework and the latter emerging from within Asian diasporic communities in the West to problematize racism and the marginalization of Asian subjects (Lee, 2015; Morris-Suzuki, 2020; Sakai, 2010). The student’s comment made me wonder: What if I actively pursued this linkage in my teaching? What would be the pedagogical implications of that? And how can I approach it critically?

Troeung (2015), in the context of teaching Asian American texts to students in Hong Kong, noted that contrary to her colleagues’ assumption about students “more interested in studying British and American literature than Asian American literature since the former was more valuable to them as cultural capital” (pp. 252–253), she found that the students were willing to engage with Asian American texts. She further stated that while the texts were read outside the original context in the United States, the students “commented on the way Asian American literature resonated with them personally in a way that canonical British and American literature did not” (p. 253). By drawing on Cheung’s (2005) notion of “pedagogies of resonance,” she developed strategies to effectively teach foreign material to students in another geopolitical context. I had a similar experience in terms of how my students responded to Japanese literature that explores sex, gender, and sexuality. To be sure, many students may be initially drawn to Japanese culture through anime and manga, which has been capitalized on by Japanese entertainment industries and government initiatives such as the “Cool Japan” policy since the early 2000s (Iwabuchi, 2015). However, as students immerse themselves in course texts, they frequently discuss the relatability of the characters’ experiences to their own, whether personal or collective. This response was expressed more strongly not only by those with Asian heritage and who were raised by Asian parent(s) or in Asia but also by other students who shared similar experiences of sexual and gender oppression.

This paper reflects on an experimental pedagogical approach I developed in the aforementioned course, where the class demographic is predominantly students of Asian heritage (mostly non-Japanese). In response to the student comment discussed above and my own research interests in embodied methodologies, my teaching intervention was epistemological rather than curatorial or content-based. Building on the intellectual movements of “Asia as method” (Chen, 2010) and “diaspora as method” (Khoo, 2019; Kim & Lee, 2019), my project aimed to move beyond the convention of studying Asian culture by referencing Euro-(North-)American theory, knowledge, and experience. Instead, it employed “embodied inter-referencing” (Park, 2019), where students discuss what they have learned from Japanese literature in relation to their own and local experiences (Cheung, 2005; Lin, 2012; Troeung, 2015) and engage in small-group interactions. At the same time, I also struggled with the question of how to decolonialize my pedagogy given that Japan, historically, has been a colonizer in Asia (Takayama, 2016, p. 28). The rest of this paper will discuss both the possibilities and challenges of this assignment based on my experience of implementing it in two sections of the course, fall of 2020 and spring of 2021; student works; and interviews with teaching assistants (TAs) from the spring of 2021 section. In sum, I will discuss the implications of highlighting student experience and peer engagement as meaningful sites for decolonial and antiracist pedagogy while teaching Japanese literature in a Canadian institutional context.

## Teaching as a nonexpert

Aligning myself with my pedagogical approach, I begin my discussion with my personal and local experience. While this is not a story of my cultural identity but rather that of my professional trajectory, my racial, gender, and linguistic identity and background have always shaped where I have found myself throughout my academic career.

I begin with a moment in 2007, when I met a female Japanese scholar who had earned her PhD in sociology in the United States and taught in a Japanese-language program at a small private university in Washington State. I visited her while doing my fieldwork research on Japanese war brides’ poetry practice, memory, and identity in Washington based on a shared research interest in the same migrant community that was the topic of her dissertation. Her trajectory revealed to me the fierce competition in the job market where one would be lucky if they were given any teaching position that provides them with some level of financial stability. While this applies to anyone who graduates with a PhD under neoliberal economic conditions, university hiring also seemed racialized and gendered. Hence, I asked myself rhetorically, what does it mean that a sociologist who is not trained to teach a language would be accepted to a language-teaching position? On what basis does this happen?

In fact, whenever I tell anyone outside the academic context that “I teach” for a living, people in Canada so often assume too hastily and inaccurately that I teach the Japanese language. Every time this occurs, I wonder whether they would hold the same assumption if I were a white person or a man. This points to another problem of language teaching that, besides being highly gendered and racialized, it has been inaccurately perceived to require less training and expertise than teaching in other disciplines and fields. People’s assumptions may reflect how women of color are underrepresented among faculty in Canadian universities. In the university where I teach, and according to the 2019 Employment Equity Report (The University of British Columbia, 2019, p. 16), women of color account for only 5% of faculty members in the Vancouver campus, while according to the 2016 census, they represent 23% of the total population in the Vancouver metropolitan area (Statistics Canada, 2021). This is in stark contrast to the university’s Asian language programs, which are overrepresented by women of color. Given that institutional racism persists in higher education in Canada (Bunjun, 2021b; Henry et al., 2017; Thobani, 2022), some people are required to use their cultural identity and heritage as resources outside or beyond their academic expertise, which happens unevenly, creating a situation where a certain group of people is forced to become more flexible and versatile by exhibiting professionalism in fields in which they are not formally trained.

Although I am a native Japanese speaker who completed my graduate degrees in interdisciplinary fields of communication and cultural studies and studied transnational migration and diasporic cultures using Japanese as my research language, I am not a Japanese area specialist. In my department, I came to teach Japanese literature and films but only as a nonspecialist in Japanese studies, literary studies, or film studies in the conventional sense. Teaching each course requires additional research and preparation as well as efforts not to be overcome by impostor syndrome. Gradually, however, I learned to incorporate my postcolonial, transnational approach, and embodied methodologies that I developed through my research into my course design. Whatever course I teach, my primary concern is to decolonize, denationalize, and diasporize them by questioning the assumption held about Japan as an autonomous sovereign entity that carries a homogenous culture and exploring antiracist and decolonial ways of teaching, learning, and sharing knowledge. This project is one tiny step toward this larger goal.

I teach Japanese literature as a “nonexpert.” I interpret this in two ways. First, I am a “nonexpert” because, as I have described, I do not fit into such a designation based on the institutional framing of what counts as professional knowledge. I make a conscious choice to use quotation marks to describe myself as a “nonexpert” in this sense because I am indeed an expert in Japanese language and culture—these are what I grew up with and where I come from. However, instead of turning my culture into a target of knowledge, what I have always done is use it as a method of research. In any case, I am also a nonexpert in another, extremely different, sense. In the context of teaching sex, gender, and sexuality in Japanese literature, students are often drawn to the course by their interests in Japanese culture as well as their lived experiences of sex, gender, and sexuality that do not conform to cisheteronormativity. In our class discussions, I often find myself being a nonexpert because as a cisheterosexual woman, I do not have a lived experience of many of the issues and priorities raised by my students. This situation creates a different classroom dynamic where my students, with their various sexual and gender identities, become experts who often expand my and their peers’ interpretations of texts.

When I say that I am no expert in the second sense, I mean it in a way elaborated by Chen (2005) in her article “Towards an Ethics of Knowledge,” where she discusses her approach to teaching multiethnic Asian American literature by “turning theory into practice and encouraging both our students and ourselves to make political commitments in the process of becoming better thinkers” (158). This involves disrupting the division between academics and activists, problematizing the traditional boundary of “expert” and “vernacular” knowledge, and reformulating pedagogical goals from ones that emphasize “mastering” the material to those that nurture the “ethics of knowledge.” The second meaning of teaching as a “nonexpert” involves my intentional reorientation of my courses’ learning objectives, whereby I invite students to the social justice work alongside me and create a space for student-centered peer learning through course materials, in-class activities, and assignments.

## Asia as method and inter-referencing

Despite the rich and influential tradition of postcolonial scholarship and its efforts to critique “orientalism” (Said, 1979), problematize “Western eyes” (Mohanty, 1988), and “provincialize Europe” (Chakrabarty, 2000), the historical legacy of the Euro-(North-)American-centric scholarship of non-Western cultures is difficult to dismantle, and it remains true that Asian cultures are so often treated as *objects* of examination, scrutiny, and judgment in the academy. The ideas, stories, and experiences of Asian people or in Asia tend to be understood as culturally specific and have little relevance outside of it, while Western knowledge is always used to comprehend and explain non-Western experiences (Chen, 2010; Chua, 2015).^1^ Consequently, when discussed in the Western institutional context, such as the university in Canada where I teach, Asian knowledge is rarely treated as references that may enhance our understanding of local experiences in locations beyond Asia. This holds true even in a classroom where students are predominantly of Asian heritage or international students from Asia. Zhang et al. (2015) pointed out a similar problem, claiming that “in many Asian educational contexts and in much research on such contexts, so-called Western theories are often adopted in an unproblematic manner, with far too little attention paid to where such ideas come from and how they are mediated in Asian educational contexts, and with insufficient attention paid to so-called non-Western educational thought and practice” (p. 3).

Teaching Japanese literature to students, the majority of whom are of various Asian heritages with diverse and shifting gender and sexual identities, I encounter three key questions. How can I develop a critical intervention into how Asian knowledge is taught as a “thing” to comprehend, describe, scrutinize, and evaluate by Western theories and languages? How can I instead turn the “resonance” that students identified in the texts into a meaningful learning resource? What strategy is effective in making the classroom a site of antiracist, antisexist, antihomophobic, and antitransphobic praxis?

While all these are large questions that cannot be fully answered in this single paper, I initiated a pedagogical intervention by building on an intellectual movement that emerged in cultural studies in Asia (or “Asian studies in Asia”) called “Asia as method” (Chen, 2010) to address some of these problems. Asia as method aims to move away from the convention of studying Asian culture by referencing Western theories, knowledge, and experiences. Instead, it examines Asia by “inter-referencing” the experiences and knowledge of other Asian countries, regions, and peoples to multiply reference points and epistemological positions in understanding globalization processes (Ong, 2011; Chua, 2015). This movement was led by Chen and Chua, who called for inter-Asia cultural studies as the site for their emerging scholarship (Chen & Chua, 2000). Given how non-Western knowledge has historically been marginalized or objectified in academia, this move is political rather than simply intellectually inspired.

Concurrently, as Zhang et al. (2015) pointed out, the notions of “Asia” as well as the dichotomy of “West” versus “East” must be “treated with particular suspicion, because of the complexities they obscure” (p. 3). In my teaching context, it is particularly important to recognize and actively examine the hierarchical power relations existing within Asia and the role Japan has historically played in marginalizing and aggressively erasing the knowledge of other Asian people through colonization. Hence, to develop a decolonial approach, it is important to “de-centre Japan,” as Takayama (2016) suggested, and “reposition (Japan) as one of the ‘nodes’ in the region through which more relevant and useful knowledge can be generated” (p. 28). Furthermore, Zhang et al. (2015) recommended that practitioners avoid “subscrib(ing) to the territorial essentialism that implies that, to do ‘Asia as method’, one has to be *living in* Asia” (p. 5). They ask, how do we factor in the geographical relocation to the West from Asia into our inquiries? Similarly, Khoo (2019) noted how “Asian diasporas in the West have largely remained ‘outside’ the intellectual project of inter-Asia cultural studies” (2019, p. 292). Extending Chen’s method, Khoo and other scholars, including Lee and Kim, who themselves are situated in Australia and Canada, further developed “diaspora as method” in studying the experiences of Asian diaspora beyond Asia, which involves inter-referencing the experiences of other Asian diasporas (Khoo, 2019; Kim & Lee, 2019; Lee et al., 2019). Not only does this allow us to examine priorities and concerns that cannot be captured through national frameworks; it also pushes Chen and Chua’s original agenda forward to “problematize Asia” (Chen & Chua, 2000; Yue in Lee et al., 2019, p. 343; Khoo, 2019, p. 292).

Chen and Hayot (2019) proposed a similar effort in their introduction to their “global Asias” project, which emerged from Asian American studies. Identifying the Eurocentric approach as the first phase and the Asia-centered approach as the second in Western scholarship on Asia, they viewed themselves as being “on the verge of an era of ‘decentred’” scholarship, which starts their inquiries from the transnational space of diasporic communities (p. viii). From this position, the boundaries between the West and Asia are viewed as fluid. As they put it, “‘Asia’ happens anytime, everywhere, and applies to everyone” (p. xi). Here again, diaspora is not treated as an identity or object of analysis but as a method and practice to study local experiences (Yue in Khoo, 2019; Lee et al., 2019). Ultimately, the inter-referencing method is about “multiplying our conceptual resources” rather than “restricting them to one part of the world” (Zhang et al., 2015, p. 7).

## Pedagogical intervention and research methodology

My project builds on these efforts but primarily with pedagogical goals in mind and invites students to practice inter-referencing by using their own personal and collective experiences as resources to discuss literary texts. This approach was inspired by the ethical and critical pedagogical practices of several scholars before me (Chen, 2005; Cheung, 2005; Lin, 2012; Troeung, 2015), but I most directly adopted Park’s (2019) strategy of “embodied inter-referencing,” which she developed in a course on institutional racism in a university in Australia in a classroom that includes domestic and international students of East, Southeast, and South Asian backgrounds. Park applied Chau’s (2015) method of “inter-referencing” and actively used the autobiographical narrative to “clarify, critique, and expand the parameters of theoretical concepts” from relevant fields of study (271). In her reflection, she discussed how this approach enabled students to become “participant(s) of collective learning and co-creator(s) of new knowledges” (281) and allowed the class to foster an “inclusive, interactive, and mutually respectful learning space” (271). While students in my course had always cited their personal experiences in informal class discussions, I adopted Park’s practice of “embodied inter-referencing” in a specific assignment to center students’ experiences and narratives more explicitly. This assignment, called the “Peer-Engaged Embodied Reflection Journal,” replaced a more traditional, closed-book midterm exam and was partly a response to the shift to online teaching during the pandemic but largely to address the questions and problems I have discussed thus far.

This paper was based on a classroom study I conducted in the fall of 2020 and the spring of 2021 and reflected on the effectiveness of this assignment in fostering a space to address the existing institutional and epistemic racism at university. To do so, I conducted a qualitative analysis of written journal entries submitted by 12 students who consented to participate in this study and semi-structured interviews with two TAs to understand their grading experience and explore their perspectives on the assignment as graders. These data were collected during and after the spring of 2021. Part of my discussion was also based on the end-of-term anonymous survey conducted in the fall of 2020, when the assignment was first implemented, which sought students’ input into the assignment in comparison to the traditional midterm exam. I also used my experience in implementing and grading the assignment as an instructor.

After describing this assignment in detail below, I discussed the students’ work based on two types of assignment outcomes: decentering Western concepts and decentering Japan. To quote students’ written work, I used full names for those who preferred to use their real names to fully acknowledge their authorship. Meanwhile, I used pseudonyms for those who preferred that their names be disguised. Gender pronouns were based on the preferences they specified in their display names on the online teaching platform. I used “they” for participants who did not explicitly specify their pronouns.

## Peer-engaged embodied reflection journal

The Peer-Engaged Embodied Reflection Journal required students to post four journal entries on the discussion board at four deadlines dispersed throughout the term and reply to their peers’ postings. For this assignment, students were assigned into groups of 20–25 and worked with the same group throughout the term. The assignment guidelines provided the following prompts to help students start their reflections: *Among the texts you read in the assigned period, which character, experience, idea, or passage does strike you the most? Why is it important to you? Why is it disturbing? How does it support, challenge, or change the way you understand your own identity or experience (or experiences of others around you)? Does it give you a new language to articulate your local experience? Does what you learned from the text have any implications for your present and/or future life?* A successful entry would discuss one specific aspect of the text and use it as a “method” to understand their local experience. This was followed by peer interactions where students inter-reference each other’s experiences by commenting on what part of their peer’s post they consider relatable or refreshing and how it supports or changes the way the student understands the text or their own identities and experiences.

The use of narrative sources from students’ experiences and their immediate lifeworld involves different levels of risk, and as an instructor, I needed to approach this carefully, especially when this becomes a required component of a graded assignment. In fact, one of the challenges my TAs and I faced while grading the students’ outputs was how to ensure that the marks they received were not based on the degree to which they made themselves vulnerable by sharing their personal experiences, or worse, inadvertently divulging their identities. We never completely resolved this concern, but I addressed this by clarifying in the assignment guidelines that students were not expected to write about things they feel uncomfortable sharing with others and ensuring that they could instead discuss issues happening around them in their local communities or the larger society in which they were situated. I also made our assessment criteria transparent and ensured that they were based not on the content of the narrative they shared but rather on their critical engagement with the chosen aspects of the text and the effectiveness of the link and relevance they established between the text and their local experience. In addition, “ethics” was included as an assessment criterion to encourage students to perform “ethical proofreading” before posting any comments to the discussion board so that their language is respectful of their peers’ diverse identities and experiences.

As a useful strategy to address the concern regarding the use of personal experience, one of my TAs, Aydin Quach, who was also a student in the course in the previous term, commented,

I think I had to put in a lot more care, because sometimes...there were some students who wish to talk about their own experiences in terms of their own sexuality. So, I needed to be aware that my grading had to somehow acknowledge that they are taking the risk and that risk should be...not just acknowledge(d) but (also) rewarded. That is how we cultivate a safe space. (Personal communication, May 14, 2021).

Aydin emphasized the importance of the manner in which we write our feedback on student works so that grading does not become “reductive” of students’ experiences. He further shared that one of the students commented that the “TA felt like a safe space” and that many students enjoyed the “opportunity to engage with the TA” throughout this assignment. I also noticed how my mode of writing shifted when providing feedback on this assignment. Besides providing constructive comments that focus on the technical dimensions of analysis and argument, I also often found myself directly responding to students’ stories and reciprocating with my experiences of being a Japanese woman in Japan or Canada and of gender, sex, and sexuality. I often began my feedback by expressing gratitude for sharing their stories for peer learning.

## Decentering Western concepts of sex, gender, and sexuality

While most students are already well-versed in Said’s critique of Orientalism by the time they attend my class and often find the texts personally relatable, it is quite challenging, especially early on in the term, to shift their mode of critique and use the texts as resources for a new language to articulate their lifeworld and experiences. Instead of removing the Western concepts of sex, gender, and sexuality or trying to replace them with their “Japanese counterparts” in our discussion (which would be simplistic and problematic given the already complex and hybrid nature of any language), I often instigated class discussions by intentionally using familiar Western concepts and encouraging students to find the *excess* of the experiences expressed in the texts that these concepts or frameworks cannot fully capture.

One of the classical texts we read in the course was *Torikaebaya monogatari* (The Changelings, or literally, The Tale of “Oh, If I Could Only Exchange Them!”) from the Heian period (794–1185) by unknown author(s). The story involves two siblings from an aristocratic family who are born as a girl and a boy and act like the opposite gender according to the norm of the Heian court. While the siblings have different mothers, they resemble each other so much, and the father, *sadaijin* (Minister of Left), decides to swap their genders so their inclinations fit the rigidly gendered roles in the court. The character whose assigned sex at birth is female occupies a male position called Chūnagon, and the one whose assigned sex at birth is male starts to serve a princess as Naishi no Kami, a female officer. In our class discussion, I would ask, “Are female Chūnagon and male Naishi no Kami ‘transgender’?” Another premodern text we read was a set of excerpts from Ihara Saikaku’s *Nanshoku ōkagami* (The Great Mirror of Male Love) from the Edo period (1603–1867), a collection of short stories about *samurai* or merchant men involved in homosexual love. Some important characters are *kabuki wakashū* (young men) actors who are also sex workers of the floating world and earn their living by serving older male (or at times female) patrons. Here I would ask, “Can we characterize a *kabuki wakashū* as ‘homosexual’ or ‘bisexual’?” Among modern texts from the Meiji and Taisho periods (1868–1912 and 1912–1926, respectively), we also discussed a male protagonist in Tanizaki Jun’ichirō’s short story “The Secret” (1911), who seeks pleasure by wearing a woman’s *kimono* and makeup and ventures into Tokyo’s nighttime entertainment district. While he is from Tokyo and is familiar with the space, the costume enables him to reexperience this familiar environment. But then, I would ask, “Could we describe the protagonist as a ‘cross-dresser’?”.

What surprised many students the most in our discussion of the pre-Meiji texts is how references to same-sex love, sex work, or sex in general are normalized or at least not tabooed in literature. However, students found it quite unsettling to apply the Western concepts of sex, gender, and sexuality in describing these characters because they hardly self-identify by these available labels and feel uncomfortable assigning any identity externally when it is unclear from the texts how they perceive themselves. For example, while the story is narrated primarily through the perspective of the siblings, the title of *Torikaebaya monogatari*, The Story of “Oh, If I Could Only Exchange Them!” expresses the father’s wish for his children to conform to gender norms as well as the rigidness and fixity of the norms of the Heian court. Early in the story, the main characters’ genders are swapped by the father, who rationalizes his unfortunate circumstance by citing the “karma” or bad deeds he committed in his previous life. Some students felt that the term “transgender” does not accurately describe the siblings’ identities and experiences because they themselves do not initiate the act of cross-dressing and their performance of opposite gender roles. Similarly, the students commented that *wakashū*’s sexuality seems produced by the structure and demands of the floating world rather than the characters’ individual sexual identities. For many students, the protagonist of Tanizaki’s story is disturbing. In one of her journal entries, Gitanjali Madan found it problematic how this “heterosexual, cis male-identified thrill seeker, uses cross-dressing as a tool for his exploration of the opposite gender.”

In fact, most stories that directly address sex, gender, and sexuality used in this course, including women’s writings from the time of the women’s liberation movement of the 1970s covered later in the term, are not necessarily “empowering” in ways that are expected by Anglo-European modern(ist) readers, “who prefer to entertain the possibility of escape, revolution, and cure” (Orbaugh, 1996, p. 154). Protagonists often do not serve as role models, and stories usually do not have a positive or hopeful closure. However, because many course readings are intentionally “writte(n) *against a background of* (cisheteronormative) patriarchal control” (ibid. p. 123, emphasis in the original), they become meaningful sites of gender and sexual politics in our class discussions. Furthermore, the reflection process, when facilitated with care and nuance, creates a highly interactive and even therapeutic space where characters that do not conform to societal norms become useful references for students to articulate their experiences in different ways than Western concepts allow them to do. For example, in her reflection of *Torikaebaya monogatari*, Laura writes,

(The story) presented a fluid view on gender and sexuality that forced me to adapt my way of thinking. Western gender and sexuality politics invite us to apply as many and as precise of labels as we can to ourselves and other people, and so it becomes confusing to read these characters whose love, gender sex seem to overlap and shift around different categories...This is not to dismiss identities of gender and sexuality with which people do choose to identify which can be empowering, but to simply consider a different way of thinking.

Discussing the same story, Denise (they/them) felt “most connected” to the female Chūnagon as a person “who is assigned the female gender at birth but live as a different gender.” Reflecting on a moment in the story when the character is sexually assaulted by a male character and Chūnagon’s “true” sex is revealed, Denise “felt a sense of terror as (they) imagined what it would be like to be forced to ‘revert’ back to a gender that (they are) not.” But it is not just Chūnagon’s experience of gender that makes the text relatable to Denise. They found the father’s belief in karma interesting and relatable as they “grew up in a culture and household of Buddhist beliefs” where their parents also mentioned “transgressions done in (their) previous lives” to lament Denise’s gender and sexuality.

At times, the texts do provide an alternative model for students to express their identities and experiences. Reflecting on the protagonist of “The Secret,” Amelia Parkin comments,

His experience with cross-dressing gave me a framework to express my desire to do something similar in a way that drag culture never has. His complete confidence in his gender identity, in that he does not question it even as he enjoys dressing as a woman, allowed me to do some introspection and realize that I am in a similar situation. I have always wanted to be treated like a man in society, specifically in academia or in times when men have approached and annoyed me because I am visibly feminine...his text may inspire me to play with my own gender expression.

However, like Gitanjali above, Amelia also identified a problematic aspect of the protagonist, especially around “the narcissistic way (the protagonist) treats” a female side character in the second half of the story. Her comments demonstrated her ability to *selectively* appropriate the character’s act to imagine an alternative gender expression.

Overall, the journal assignment offered students an opportunity to multiply reference points to discuss sex, gender, and sexuality, and this was true for any student with varied degrees of familiarity with Japanese literature and culture. Aydin eloquently summarized the value of adopting Asia as method in the course assignment:

(Students’) home culture or the culture that is the most comfortable expressing themselves in...can be enriched by cultures outside of their normal lived experiences. That is mostly for the students who are not of Japanese heritage or may not be readily exposed to Japanese literature. (But) there are a few students (who) are exposed to Japanese culture at home through parents of Japanese heritage, and I find that in their reflection journals, they found that the text enriched or elucidated more about their culture than their...parental figures or elders could express. So I think there are still that...multiplying frames of understanding that applies even to native or heritage learners.

## “American Hijiki”: decentering Japan

Given that the majority of my students in the course were of non-Japanese Asian heritage(s) or background from countries or regions that have been historically affected by Japanese (neo)colonialism, one of my priorities was to create an anticolonial space in my classroom by actively discussing Japan’s colonial history and creating a safe space for students to discuss the ongoing impacts of Japanese colonialism. In this subsection, I will discuss how the journal assignment effectively empowered the class to discuss race, sex, gender, and sexuality in the context of colonialism/war/occupation while decentering Japan and instead centering students’ embodied experiences, focusing on the students’ work on Nosaka Akiyuki’s short story “American Hijiki” (*Amerika hijiki*, 1967/2005).

Nosaka’s award-winning semiautobiographical novella “American Hijiki” (1967/2005) features Toshio, a middle-aged married man who survived the U.S. air raids during the Pacific War as a youth and the subsequent U.S. military occupation as a pimp serving American soldiers stationed in Kobe. The narrator moves back and forth between flashbacks of Toshio’s experience of the (post)war periods and his present life in Tokyo, where he reluctantly receives Mr. and Mrs. Higgins from the United States as guests. His recollection is centered on the sudden and outright paradigm shift caused by Japan’s defeat. A Chinese teacher is replaced with an English teacher at school, “American and English Devil-Brutes” are now “gentlemen” whom the Japanese should look up to, and the U.S. aircraft that used to drop bombs during the war are now supplying “treasure cans” containing sugar, a symbol of luxury during the war. Included in these treasure cans is “stringy, black stuff,” which only looks like the seaweed *hijiki* in the eyes of Japanese people who have never seen black tea before. The title of the novella, “American Hijiki,” epitomizes Toshio’s (and the author’s) embodied memory of the bitterness of the black tea and Toshio’s relationship with Americans and American culture. Nosaka’s depiction of the physicality of the large American male bodies conveys Toshio’s gendered inferiority complex that developed in the aftermath of the war. The emasculation of Japanese men is presented most strikingly in the climax scene set in the present time where Toshio invites Mr. Higgins to a live sex show to present the “numbah one penis, the pride of Japan” (Nosaka, 1967/2005, p. 465) and “bring (Higgins) to his knees” with awe (Nosaka, 1967/2005, p. 463), resulting in complete failure when the male sex worker suffers from sudden impotence in front of a white male ex-officer.

Written from the perspective of a Japanese protagonist who survived the war and the ensuing U.S. occupation, “American Hijiki” decenters the Western (or American) worldview and perspective of transpacific history. At one level, it troubles, if not subverts, America’s “good war” narrative produced in the mid-twentieth century by the U.S. “empire for liberty” in the context of the Cold War (Yoneyama, 2017, p. 472) by vividly portraying the everyday experience of war trauma and how the U.S. occupation has emasculated Japanese men particularly and Japan generally. At a broader level, however, as student Jasmine Snau pointed out, the title “American Hijiki” and the Japanese people’s encounter with an unknown Western product (black tea leaves) with no equivalent Japanese word “encapsulates the discordance of culture.” It reveals linguistic and cultural untranslatability, the presence of a paradigm that cannot easily be assimilated into another; Western culture becomes an alien rather than the universal self that literally comes from the sky. Furthermore, as Jasmine noted, the way Mr. Higgins comfortably moves around in the “Americanized Japanese space,” combined with how Western products are dropped from U.S. aircraft just like the bombs that defeated Japan, signifies the “power imbalance” between the two paradigms.

In “American Hijiki,” Toshio’s “inferiority” is also constructed through his English pronunciation, which has a heavy Japanese accent and is textually expressed through the romanization of Japanized English. Speaking from her experience as a Japanese person who speaks English fluently, Suzu related this aspect of the text to her own encounter with the existing language hierarchy in Japan:

English and America-worshipping are still common in Asia and I strongly felt that in my life back in Japan. In high school, my friends over-praised my English skills and were very amazed by how I spoke English like a foreigner/Westerner (without any Japanese accents). They often thought I was “smart” for being able to speak English, but in reality, I was just a returnee student. However, bilingual students who spoke Chinese, Korean, or any other non-Western languages were definitely underrated and not praised as much.

Given Japan’s own status as a former colonizer and occupier in Asia, I grappled with the following question: How can I engage students with Nosaka’s story without reproducing Japan’s self-victimizing narrative and downplaying the country’s own history of colonialism and racism in Asia? As Yoneyama (1999) pointed out, Japan’s self-victimizing narratives were formed in the immediate postwar period around U.S. air raids, and the atomic bombings of Hiroshima and Nagasaki ultimately contributed to the forgetting of Japan’s imperial aggression and victimization of Asians and Pacific Islanders. Is it possible at all to effectively discuss the “critical differences, the historical specificities and the asymmetrical positions that distinguish Japan from its neighboring countries” using this text (Yoneyama, 1999, p. 12)?

What I did to address this problem in the past was to remind students of Japan’s history of colonialism and the country’s failure to sincerely address its colonial and war crimes from the end of WWII to the present. I would normally discuss this in previous lectures in relation to other modern texts. I would also point to a future reading by Yū Miri, where we discuss the history of Zainichi Koreans and the ongoing effects of Japan’s colonialism on the characters’ everyday experience of family, gender, and sexuality. In addition, I would briefly introduce other courses taught by a department colleague that directly focus on colonialism and racism in Japanese literature. But more importantly, and perhaps in a much more effective way, the journal assignment allowed students to discuss the multilayered power relations shaping the transpacific world directly in their analysis of Nosaka’s story by inter-referencing their personal and family experiences or events in their respective immediate environment.

For example, Denise, who is of Taiwanese descent, wrote in their journal entry that the text stood out for them because they “saw a lot of (their) grandfather in the story.” After describing how much of their culture has been influenced by Japan’s colonialism in the late nineteenth century and the first half of the 20th, they wrote that the passages describing how the English teacher pronounced *thank you*, “San-Q,” reminded them of their grandfather, who pronounced the phrase in the same exact way. They wondered if this was also a haunting effect of the colonial Japanese schooling during the colonial period and its lasting influence on him. Another student from South Korea expressed their mixed emotions reading the text as it is a story told through the voice of a person from the colonizer and perpetrator nation. Other students further extended their discussions of the text to other experiences of colonialism and war by referencing the U.S. colonization of the Philippines or the Vietnam War based on their family backgrounds in affected countries in Asia. Student reflections such as these destabilize the West/East, victimizer/victim, and influencer/influenced dichotomies and reject a simplistic narrative of Japan’s victimization.

Some unintended consequences of this assignment from the context of the “American Hijiki” discussion were how students related Toshio and his wife Kyōko’s “inferiority complex” manifested in their constant attempts to please Mr. and Mrs. Higgins and students’ own migratory experiences of coming to North America as Asians; feeling “inferior” to other children because of their language, race, and culture; and finding themselves constantly comparing themselves to white children. This thread of discussion ended up being quite vibrant, inviting responses from peers with similar experiences as well as a white American student who grew up in a predominantly white town, who found the discussion eye-opening. The way in which race intersects with gender and sexuality was also an important discussion topic. Reflecting on how Japanese sex workers are displayed in front of Mr. Higgins, students discussed how such portrayal remains relevant today given the prevalence of Internet images and comments that put East Asian sexuality under the Western male gaze. One student further made a reference to anti-Asian racist crimes that became palpable in response to the COVID-19 pandemic and, particularly, the mass shooting of sex workers in Atlanta, Georgia, which happened in March 2021, immediately before the discussion. The student discussed how the crime was enabled by both white supremacy and misogyny just like how the live sex show presented to Higgins is both racialized and sexualized.

In sum, students appropriated the language of “American Hijiki” to articulate other experiences of colonialism, occupation, war, race, and sex as they decenter both the West and Japan by foregrounding their diverse and immediate experiences.

## Conclusion

Overall, students’ feedback on the Peer-Engaged Reflection Journal assignment was positive. Based on the anonymous student survey conducted at the end of the first term, 29 of 33 students (88% [41% response rate]) stated that they preferred the journal assignment to the traditional midterm exam from previous iterations of the course. What students appreciated the most about the new assignment was the opportunity to relate the texts to their own experiences and the extent of meaningful and thoughtful peer engagement it generated while still being challenging throughout. This was particularly important as the students took the course remotely, did not have an opportunity to meet their peers face-to-face, and, depending on the country or region they were located during the term, had generally limited in-person interactions in their life. Ashley Robinson, who, like Aydin, was a student in the course when the assignment was first implemented and worked as a TA for the course in the second term, observed, “Students had a really broad range of experiences that they brought to that assignment. It managed to allow all of them to contribute those experiences that were extremely different” (personal communication, May 14, 2021). In addition, Ashley commented that what was unique about this assignment was how it “assessed (students’) ability to look at the text as functional thing” rather than something that is “distant” from their lives and concerns.

While this paper focused more on the assignment’s impact on decentering Western knowledge and Japan in class discussions, one crucial contribution that the students made through their engagement with the assignment was solidarity building. In each group, the students managed to build a community of care using respectful and empathetic language, commenting on how their peers’ embodied inter-referencing further enabled them to reflect on the text and on their experiences in a new light. Interactions took place not only between those who shared the same identity or similar experiences but also across differences. This was incredibly crucial in the pandemic period, where anti-Asian hate crimes surged on campus and beyond. In fact, the 2019 Academic Experience Survey (Burnham et al., 2019/20) conducted at my university before the COVID-19 outbreak reported that “international students are more likely to report experiencing racial discrimination (43%) than domestic students (35%)” and that the “majority of undergraduate students (57%) continue to experience some form of discrimination on campus, most commonly due to race/ethnicity (36%) or age (25%)” (p. 29). These numbers are considerably high given that only 42% of the samples were undergraduate students with a Caucasian ethnic background. This number would be even smaller if students with mixed heritages were excluded. While the classroom is only one part of students’ lives, it is imperative to use every opportunity to build a community of care as well as foster anticolonial and antiracist solidarity.

## Footnotes

1 I distinguish “Western” from “non-Western” as an epistemological rather than a geographical difference, following Orion Noda’s (2020) definition and recognizing the history of colonialism and Eurocentricism that has conditioned the global production, dissemination, and application of academic knowledge. As I discuss below, I approach this dichotomy critically to recognize the existing inequality and colonial power relations within Asia.
